# Beyond the spike: Functional (dissociative) seizures as a key to holistic attitudes in seizure disorders

**DOI:** 10.1111/epi.18651

**Published:** 2025-09-23

**Authors:** Mahinda Yogarajah, Barbara Dworetzky, Josemir W. Sander, Angel Aledo‐Serrano

**Affiliations:** ^1^ Department of Epilepsy UCL Queen Square Institute of Neurology London UK; ^2^ Chalfont Centre for Epilepsy National Institute for Health and Care Research University College London Hospitals Biomedical Research Centre London UK; ^3^ Department of Neurology, Edward B. Bromfield Epilepsy Center Brigham and Women's Hospital, Harvard Medical School Boston Massachusetts USA; ^4^ Epilepsy and Functional Neurological Disorders Unit Vithas Madrid University Hospital Madrid Spain

**Keywords:** epilepsy, epileptic seizures, functional/dissociative seizures

## Abstract

Epileptologists can improve outcomes through follow‐up and coordination of care for patients with functional seizures. Epileptic and functional seizures share some mechanistic overlap involving interoceptive, emotional, and stress dysregulation, and disorders of agency and perception. Similar psychiatric and neurological comorbidities occur at comparable rates in both functional and epileptic seizure populations. A holistic, biopsychosocial approach benefits all seizure patients and reflects modern models of epilepsy care.

## 1 INTRODUCTION

People with functional or dissociative seizures (FDS) are at least as common as those with multiple sclerosis,^1^, ^2^, ^3^ with an incidence ≈3/100 000/year and prevalence of 24–109/100 000. However, these values likely underestimate the true frequency, given that most studies restrict case ascertainment to video‐electroencephalographic (EEG) telemetry‐confirmed diagnoses, which is the minority in routine practice. In a typical seizure disorder clinic, more than one fifth of people attending for the first time have FDS and not epileptic seizures (ES).^4^ Beyond diagnostic considerations, however, epilepsy specialists and funding agencies are often reluctant to engage clinically and academically with this disorder. FDS are typically dismissed as outside the remit of epileptologists. Arguments against involvement include the absence of hypersynchronized neuronal discharges in FDS and the perceived greater psychiatric complexity of these individuals.^5^ The result is a significant gap in care and research funding.^6^ This neglect has clinical and economic consequences, as people with FDS incur health care costs,^7^ morbidity levels,^8^ and premature mortality rates^9^ similar to those associated with ES.

We highlight the inherent contradictions in these arguments (Table 1). We advocate that seizure specialists should take a more central clinical and academic role regarding FDS, ideally within a multidisciplinary framework.

**TABLE 1:** Rationale for greater epileptologist engagement with functional seizures.

Domain	Rationale
Prevalence & burden	Functional seizures are common in seizure clinics and carry similar morbidity, mortality, and health care costs as epilepsy
Clinical responsibility	Neurologists diagnose FDS and are well placed to optimize ASM use, assess new symptoms, and coordinate care
Treatment impact	Even brief neurologist‐led interventions (e.g., motivational interviewing, outpatient follow‐up) can reduce seizure frequency and improve outcomes
Comorbidity overlap	High rates of psychiatric and neurological comorbidities (e.g., PTSD, ASD, TBI) are present in both FDS and epilepsy
Mechanistic convergence	Shared network dysfunction, stress biology, autonomic and interoceptive pathways, and abnormalities of perception link FDS and epilepsy
Therapeutic cross‐benefit	Neurobehavioral therapies developed for FDS may benefit epilepsy patients, suggesting overlapping treatment targets
Mixed diagnosis	Many patients have both epileptic and functional seizures, requiring integrated and nuanced management
Changing models of epilepsy	Network‐based and biopsychosocial models of epilepsy align with FDS frameworks, challenging rigid spike‐based definitions
Health system precedent	Other specialties (e.g., gastroenterology, movement disorders) take active roles in managing functional conditions

## 2 A ROLE BEYOND MAKING A DIAGNOSIS

Because people with FDS present to seizure disorder clinics, specialists in these settings have an established diagnostic role. However, the idea that this is their only role, while management and research are left to others, is at odds with other functional disorders. Although specialized epilepsy‐led multidisciplinary services for FDS are limited, there exist gastroenterology‐led and coordinated multidisciplinary services for irritable bowel syndrome.^10^ Even within neurology, in an international survey of 864 movement disorder specialists, 60% considered that their primary responsibility was not only to provide a diagnosis, but also to coordinate interdisciplinary management of functional movement disorders.^11^ This is in contrast to the opinions expressed by epileptologists in a comparable study where only a minority held similar views.^12^

In individuals with FDS alone, seizure specialists play an essential research and clinical role. Even the minimum of care, that is, the provision of follow‐up appointments, has indirect benefits. Seizure specialists are best placed to wean inappropriately prescribed antiseizure medications (ASMs), assess new seizure manifestations, and manage common comorbid neurological conditions such as migraine. Optimizing the management of these conditions typically improves the frequency of the FDS.^13^ More implicit benefits arise from the finding that people with FDS view their seizures as biological and psychological.^14^ Continuing neurology input leads to increased engagement with psychological/psychiatric services, resulting in improved outcomes. In the CODES (Cognitive Behavioral Therapy Versus Standardized Medical Care for Adults With Dissociative Non‐Epileptic Seizures) study, which is the largest study of cognitive behavioral therapy (CBT) in FDS, there was no difference in seizure frequency when comparing standardized medical care alone and standardized medical care with CBT.^15^ This may partly have been because standardized medical care consisted of structured neurology input, alongside psychiatric follow‐up.^16^ A smaller Australian study showed significant health economic savings in individuals with FDS when there was continuing neurology follow‐up.^17^

With appropriate resources and training, interested seizure disorder specialists could provide more direct care for these individuals. This could range from helping to coordinate multidisciplinary care among psychiatrists and psychologists already embedded within a seizure disorder service^18^ to delivering brief but impactful interventions. A randomized controlled trial showed that a neurologist with minimal training and using a single 30‐min session of motivational interviewing doubled psychotherapy uptake. Moreover their interviewing also led to a measurable reduction in seizure frequency, even among those who never accessed therapy.^19^

Lastly, seizure disorder specialists should also engage with this condition beyond diagnosis, because of the frequent co‐occurrence of both epileptic and functional seizures.^20^ The challenge is not only to distinguish between the two seizure types, but to manage them holistically, especially given that one is primarily treated pharmacologically with ASMs, and the other requires a multidisciplinary, nonpharmacological approach.

Seizure specialists have the expertise to make a diagnosis of FDS, and general psychiatrists are frequently either skeptical regarding the diagnosis or lack confidence and experience in managing it.^5^, ^21^ It is therefore essential that seizure specialists step forward to help coordinate the management and deliver care to these individuals.

## 3 BIOLOGY BEYOND HYPERSYNCHRONIZATION

Despite the disinterest in FDS, epilepsy specialists readily engage clinically and academically with other types of seizures in individuals without epilepsy, such as provoked seizures caused by other conditions. The argument offered is that there are common seizure mechanisms, such as neuronal hypersynchronization, so that the study of provoked seizures can help to understand how external and/or internal factors modulate epileptic seizures. However, FDS also provide a window for understanding how other, more distal mechanisms may be relevant in epileptic seizures. Research into the neurobiology of FDS has shown that stress biology, autonomic and interoceptive dysfunction, and emotional dysregulation can modulate FDS.^22^ These biological systems are also relevant to people with epilepsy, where it is accepted that autonomic dysfunction has a role in sudden unexpected death in epilepsy.^23^ Psychological stress can exacerbate ES, and emotional dysregulation plays a role in psychiatric comorbidity and quality of life.^24^ The overlap is reflected in contemporary mechanistic models of functional neurological disorders and FDS. These disorders can be conceptualized within the framework of abnormal functioning of relevant brain networks, such as the default mode, salience and attentional networks, alongside psychosocial factors.^25^ This network‐based perspective aligns with recent shifts in epilepsy research, where the disorder, and especially interictal comorbidity, are now increasingly viewed as a disruption across interconnected brain systems.^26^, ^27^ In acknowledging the possibility of mechanistic overlap, it follows that treatment for FDS may offer some benefit to those with epileptic seizures. A prospective trial of neurobehavioral therapy demonstrated that a structured, multimodal psychotherapy originally developed for FDS could also significantly reduce seizure frequency in people with epilepsy during the active treatment phase.^28^ The epilepsy cohort experienced a 34% reduction in monthly seizure frequency during neurobehavioral therapy, with seizure freedom achieved in 74% of participants by the end of treatment. However, these gains were not sustained at the 1‐year follow‐up. These improvements occurred without changes to ASMs. They may be due to shared neurobiological mechanisms between epilepsy and FDS, involving arousal, stress, and emotional regulation, all of which are targeted in neurobehavioral therapy. Neurobehavioral therapy integrates cognitive, psychodynamic, mindfulness, and self‐regulation strategies, which may support improved seizure control through greater self‐awareness, reduction of trigger sensitivity, and enhanced stress resilience. The observed improvements suggest that such therapies warrant further exploration in epilepsy care as adjunctive treatments.

Other mechanistic overlaps have emerged through the use of long‐term subcutaneous EEG recordings in people with epilepsy.^29^ Individuals report events that are not epileptic seizures and miss events that are epileptic seizures. The most recent study of long‐term subcutaneous EEG recording in 31 individuals with epilepsy over 6 months noted that self‐reported nonepileptic events (*n* = 84) and unreported electrographic seizures (*n* = 14) both outnumbered self‐reported electrographic seizures.^30^ This raises a critical question: where do ES end and FDS begin in these individuals, most of whom would not be categorized as having "typical functional seizures." In ES, there are inherent and underexplored disturbances in perception, agency, and consciousness, concepts that fundamentally define functional seizures.^31^

**FIGURE 1:**
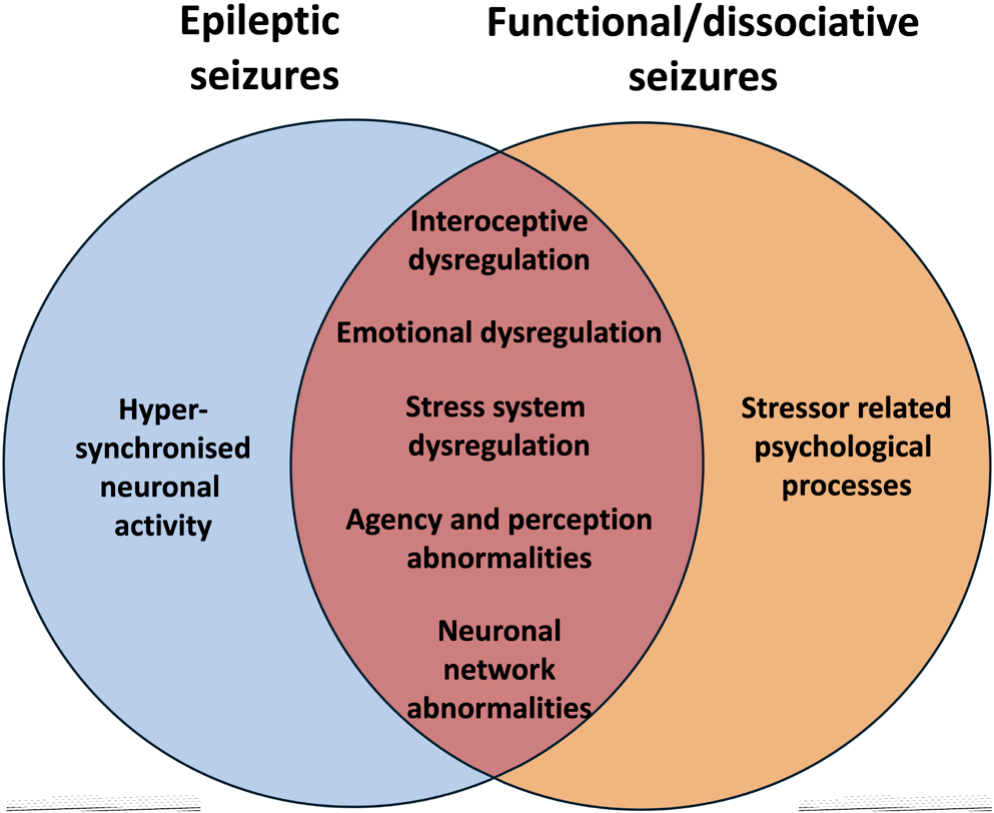
The mechanistic overlap between epileptic and functional/dissociative seizures.

Taking more of an interest in the biological underpinnings of FDS may help us understand why seizure reporting is often inaccurate in ES, and how higher order factors such as stress, attention, and emotion affect epileptic seizures. Dismissing FDS because they lack EEG spikes risks ignoring mechanisms relevant to both conditions (Figure 1).

## 4 BEYOND ASMs: NEED FOR A BIOPSYCHOSOCIAL APPROACH

Some argue that FDS are too complex because of significant psychosocial comorbidities. However, one third of people with FDS have no significant psychiatric comorbidity.^16^ In the remainder, although meta‐analyses confirm that psychiatric comorbidity is approximately 1.3 times more common in FDS than ES,^32^ there are high levels of psychiatric comorbidity in drug‐resistant epilepsy, with rates of >50% in large studies.^33^ Psychiatric comorbidities traditionally associated with FDS such as posttraumatic stress disorder and somatic symptom disorder are now recognized to be present at elevated rates (18%^34^ and 24%,^35^ respectively) in people with ES. There are complex interactions between comorbidities, including depression, anxiety, and somatic symptom disorder, that determine the quality of life in ES individuals.^36^, ^37^ Similarly, autism spectrum disorder^38^ and traumatic brain injury,^39^ which have long been associated with epilepsy, are now recognized in nearly 20%^40^ and 70%,^41^ respectively, of individuals with functional seizures. Comorbidities in both disorders have roots in a complex interplay of biological, psychiatric/psychological, and social processes. Complexity is not unique to FDS. The biopsychosocial model, long recognized as integral to the management of FDS, should be extended to those with ES, where it may likewise improve outcomes.^42^

## 5 THE WAY FORWARD

If one defines epilepsy so narrowly that only spike‐driven events fall within its remit, one must accept that this definition also fails many of those with epilepsy too, particularly those with unmeasurable, subjective, or comorbid phenomena that nonetheless define their lived experience. If this is acknowledged, then it follows that epilepsy specialists have a central role in treating what individuals experience as seizures, regardless of mechanism. FDS, especially when comorbid with epilepsy, falls squarely within the remit of an epileptologist.

Functional seizures offer valuable insights and relevant information to enhance our understanding of ES and their management. Integrating FDS into epilepsy practice and research is feasible and essential to the holistic evolution of comprehensive care and research. We urge funding agencies to include functional seizures in research agendas, and health care providers to foster a more inclusive environment that addresses the multifaceted nature of seizure disorders. Interdisciplinary training, research grants, and integrated care models are avenues to enhanced understanding, improved individual and quality of life outcomes, and lower health care costs regardless of seizure type.

## AUTHOR CONTRIBUTIONS

All authors contributed to all aspects of the article.

## FUNDING INFORMATION

M.Y. is funded by an MRC CARP award (MR/V037676/1).

Abbreviations: ASD, autism spectrum disorder; ASM, antiseizure medication; FDS, functional or dissociative seizures; PTSD, posttraumatic stress disorder; TBI, traumatic brain injury.

## CONFLICT OF INTEREST STATEMENT

M.Y. has served as a consultant providing specialist medicolegal advice regarding functional seizures. B.D. is president of the Functional Neurological Disorder Society. The other authors have no conflicts of interest to declare concerning this article. We confirm that we have read the Journal's position on issues involved in ethical publication and affirm that this report is consistent with those guidelines.
